# Correction: Pupillometry evaluation of melanopsin retinal ganglion cell function and sleep-wake activity in pre-symptomatic Alzheimer’s disease

**DOI:** 10.1371/journal.pone.0230061

**Published:** 2020-02-27

**Authors:** Angela J. Oh, Giulia Amore, William Sultan, Samuel Asanad, Jason C. Park, Martina Romagnoli, Chiara La Morgia, Rustum Karanjia, Michael G. Harrington, Alfredo A. Sadun

The Funding statement for this paper is incorrect. The correct statement is: AAS and SA were supported by the Research to Prevent Blindness, Inc. (unrestricted grant). MGH was supported by the L.K. Whittier Foundation.
